# Value of prognostic scores in antineutrophil cytoplasmic antibody (ANCA) associated vasculitis patients in intensive care unit: a multicenter retrospective cohort study from Turkey

**DOI:** 10.3906/sag-1911-86

**Published:** 2020-08-26

**Authors:** Uğur ÖZDEMİR, Ebru ORTAÇ ERSOY, Recep Civan YÜKSEL, Erhan KAYA, Gülbin AYGENCEL, Melda TÜRKOĞLU, Arzu TOPELİ, Muhammet GÜVEN, Murat SUNGUR, Neriman Defne ALTINTAŞ

**Affiliations:** 1 Division of Intensive Care Medicine, Department of Internal Medicine, Gazi University School of Medicine, Ankara Turkey; 2 Division of Intensive Care Medicine, Department of Internal Medicine, Hacettepe University Faculty of Medicine, Ankara Turkey; 3 Division of Intensive Care Medicine, Department of Internal Medicine, Erciyes University School of Medicine, Kayseri Turkey; 4 Division of Intensive Care Medicine, Department of Internal Medicine, Ankara University School of Medicine, Ankara Turkey

**Keywords:** Antineutrophil cytoplasmic antibody, vasculitis, prognostic scores, critical care, mortality

## Abstract

**Background/aim:**

There is a need for a scoring system for predicting ICU prognosis of patients with ANCA-associated vasculitis (AAV), but there are limited data on it in the literature. Therefore, we aimed to determine the scores that can estimate the prognosis of patients with AAV during intensive care follow up.

**Materials and methods:**

All adult patients admitted to the medical ICUs of 4 reference university hospitals in Turkey due to AAV activation and/or disease/treatment complications in the last 10 years were included in this study. Demographic data, treatments before ICU, the Birmingham Vasculitis Activity Score (BVAS) score at the time of vasculitis diagnosis, and BVAS, APACHE II, SOFA, and SAPS II scores at the ICU admission, treatments, procedures, and complications during ICU stay were recorded for all AAV patients.

**Results:**

Thirty-four patients were included in the study. The median age of the patients was 60 (42–70) years, and 64.7% were male. Twenty-five patients were diagnosed with Granulomatosis with polyangiitis, and 9 were diagnosed with Microscopic polyangiitis. The most common ICU admission causes were hemorrhage (85.3%) and sepsis/septic shock (67.6%). Twenty patients (58.8%) died in the ICU follow up. There were significant differences in APACHE II (P = 0.004) and SAPS II (P = 0.044) scores between survivors and nonsurvivors, while there were no significant differences in BVAS (during diagnosis P = 0.089 and ICU admission P = 0.539) and SOFA (P = 0.097) scores. APACHE II score was found to be an independent risk factor for ICU mortality (OR = 1.231, CI 95% = 1.011–1.498, P = 0.038) according to logistic regression analysis. An APACHE II score of greater than 20.5 predicted ICU mortality with 80% sensitivity and 70% specificity (AUC = 0.8, P = 0.004, Likelihood ratio = 2.6) according to the ROC curve analysis.

**Conclusion:**

APACHE II score can be used for the prediction of ICU mortality in AAV patients.

## 1. Introduction

Antineutrophil cytoplasmic antibodies (ANCA) and associated vasculitis (AAV) is a small vessel vasculitis that differs from other vasculitides with its common characteristic features. AAV is characterized by necrotizing inflammation involving predominantly small vessels and accompanied by the presence of ANCAs in serum [1]. The ANCAs, which have a significant role in the pathogenesis of the AAV, can be against proteinase-3 (cytoplasmic, c–ANCA) or myeloperoxidase (perinuclear, p–ANCA) [2]. AAV includes microscopic polyangiitis (MPA), granulomatosis with polyangiitis (GPA), eosinophilic granulomatosis with polyangiitis (EGPA), and drug-induced AAV [3]. During the clinical course of patients in the AAV disease group, the general condition of the patients may deteriorate rapidly, and admission to the intensive care unit (ICU) may be necessary due to life threatening complications [4]. These complications can be listed as pulmonary alveolar hemorrhage, subglottic obstruction, coronary artery syndromes, intestinal ischemia and perforation, crescentic glomerulonephritis and renal failure, intracranial hemorrhage, immunosuppression due to the drugs used, pneumonia and sepsis [5]. Due to this wide range of complications, the clinical outcome of the AAV patients can be highly variable and unpredictable.

An Acute Physiology and Chronic Health Evaluation (APACHE) II score, Sequential Organ Failure Assessment (SOFA) score, or Simplified Acute Physiology Score (SAPS) II can be used to determine the prognosis of patients in the ICU [6]. The activation of the AAV can be associated with more severe complications and requirements of organ support. The Birmingham Vasculitis Activity Score (BVAS), which was originally developed in 1994 (version 1) [7], can be used for the evaluation of vasculitis activation of patients. Therefore, the BVAS may be useful in assessing the prognosis of patients with AAV. The BVAS has 2 new versions, version 2 and version 3. The latest version of the BVAS (version 3) was also validated for ANCA related vasculitis [8,9]. To our knowledge, there is a limited number of studies in the literature investigating the clinical and ICU outcomes of patients with AAV [4, 10–13]. In fact, these patients constitute only a small part of the patients who are admitted to the ICU [5]. However, the management of these patients in ICUs can be quite difficult due to unexpected life threatening complications associated with acute vasculitic manifestations and immunosuppressive therapies. We less encountered this patient population in intensive care practice. For these reasons, in this multicenter, retrospective study, we planned to determine the clinical factors that can influence prognosis, the prognostic importance of the activation degree of the vasculitis (by BVAS version 3) and especially the prognostic importance of prognostic scores, which are frequently used in intensive care practice (by the APACHE II score, SOFA score, SAPS II score). 

## 2. Materials and methods

### 2.1. Patient population 

This retrospective multicenter study included patients with ANCA associated vasculitis (AAV) who needed medical ICU admission in 4 reference hospitals of Turkey. Three of these hospitals are located in Ankara province. These are Gazi University Hospital, Hacettepe University Hospital, and Ankara University İbn-i Sina Hospital, respectively. The other reference hospital is Erciyes University Hospital in Kayseri province. All patients who were diagnosed with ANCA associated vasculitis before or during ICU stay, who were older than 18 years of age, and who needed to be followed up in medical ICU between January 1, 2008 and January 1, 2018 were included in this study. For AAV patients with more than one ICU admission, only those who were admitted to the ICU for the first time were included in the study. The AAV diagnosis of all patients included in this study was consistent with the criteria of the European Medicines Agency vasculitis classification [14]. This study was approved by the Local Ethics Committee of Gazi University (date: March 26, 2018 and issue: 234).

### 2.2. Clinical information about patients

The following data were obtained from AAV patients: demographic data, test results of ANCAs (by immunofluorescence), pathological data if available, duration from diagnosis of AAV to ICU admission, the class of AAV diagnosis according to the European Medicines Agency vasculitis classification, BVAS score at the diagnosis (BVASdg), and BVAS score at the ICU admission (BVASicu), length of ICU stay, mechanical ventilation requirement, the main reasons of the ICU admission, Glasgow Coma Scale (GCS), hemodialysis and continuous renal replacement therapy requirement, AAV related organ involvements, immunosuppressive treatments and doses of the drugs used, plasmapheresis requirements, routine laboratory tests, infections, sepsis, septic shock, and related microorganisms. The following scores were also obtained from the patients 24 h after ICU admission; Acute Physiology and Chronic Health Evaluation (APACHE) II score, Sequential Organ Failure Assessment (SOFA) score, Simplified Acute Physiology Score (SAPS) II. Patients diagnosed with AAV within the last 30 days before ICU admission or during ICU stay were considered as newly diagnosed patients [10]. On the other hand, the case of patients who had more than 30 days between AAV diagnosis and ICU admission and were admitted to ICU with new vasculitis involvement was considered as a relapse.

### 2.3. Statistical analysis

Statistical analysis was performed using the IBM SPSS statistical software package version 22 (IBM Corp., Armonk, NY, USA). Because this study group was small, continuous variables were accepted as nonnormally distributed and were described as median and interquartile ranges. Categorical variables were presented as frequencies and percentages. Patients were divided into 2 groups as survivors and nonsurvivors. The Mann-Whitney U test was used to compare the continuous variables between the 2 independent groups. The Chi-square test was used to compare categorical variables. Among the variables that indicated statistically significant differences regarding mortality, the logistic regression analysis was performed to determine the variables that were independently related to mortality. After the determination of independent risk factors for mortality, ROC (Receiver Operating Characteristic) curve analyses were performed. 

## 3. Results

Thirty-four patients were included in the study. The median BVAS value at the diagnosis of AAV was 23. c–ANCA was determined in 24 (70.6%) patients, and p–ANCA was determined in 12 (35.3%) patients. Twenty-five (73.5%) patients were diagnosed with GPA, and 9 (26.5%) were diagnosed with MPA. The demographic and clinical data of the study patients and their statistical differences according to mortality are presented in Table 1. Only 3 patients were diagnosed with AAV during ICU stay, but all of the others had been diagnosed with AAV before ICU admission. According to table 2, there were not differences between survivor and nonsurvivor patients in terms of disease related features, except than presences of septic shock. The respiratory failure due to hemorrhage and sepsis/septic shock were the main reason of the ICU admission (Table 2). Twenty patients (58.8%) died during ICU stay. Twelve patients died due to septic shock (60%). Four of them died due to massive hemoptysis and respiratory failure (20%). Two of them died due to hemorrhagic shock (10%). One of them died due to cardiogenic shock (5%). One of them died due to resistant pulmonary edema and respiratory failure (5%). Some laboratory results of the survivors and nonsurvivors are shown in Table 3. Statistically significant differences were found in clinical prognostic scores (APACHE II and SAPS II) between survivors and nonsurvivors. Also, immunosuppressive therapies applied before ICU admission and during ICU stay in survivors and nonsurvivors are presented in Table 4. There was no difference between survivors and nonsurvivors in terms of immunosuppressive therapies. New–onset infections and related pathogenic microorganisms in AAV patients during ICU stay are listed in Table 5. APACHE II score was found to be an independent risk factor for ICU mortality when the logistic regression analysis was performed between APACHE II, SAPS II, SOFA scores, BVAS at the diagnosis, serum ALT level and blood platelet count (Table 6). The cutoff value of the APACHE II score for prediction of ICU mortality in AAV patients was found to be 20.5 with 80% sensitivity and 70% specificity (AUC = 0.800, P = 0.004, LR = 2.6) according to Receiver Operating Characteristic (ROC) analysis (Figure).

**Table 1:** Demographic and clinical data of AAV patients.

	Parameters	All patients (n = 34)	Survivors (n = 14)	Nonsurvivors (n = 20)	P value
	Age (Years)*	60 [42–70]	63 [47–70]	57 [40–70]	0.592
	Sex, M, n (%)	22 (64.7)	9 (64.3)	13 (65)	0.966
	Smoking, n (%)	10 (29.4)	5 (35.7)	5 (25)	0.506
Comorbidities	Chronic kidney disease, n (%)	14 (41.2%)	6 (42.9)	8 (40)	0.870
	AKIN stage 1/2/3, n (%)	2 (14.3) / 1 (7.1) / 11 (78.6)			
	Essential hypertension, n (%)	11 (32.4%)	6 (42.9)	5 (25)	0.396
	Coronary artery disease, n (%)	6 (17.6)	3 (21.4)	3 (15)	0.634
	Heart failure, n (%)	5 (14.7)	3 (21.4)	2 (10)	0.362
	Rheumatoid arthritis, n (%)	3 (8.8)	2 (14.3)	1 (5)	0.355
	Type 2 diabetes mellitus, n (%)	2 (5.9)	1 (7.1)	1 (5)	0.931
	Hypothyroidism, n (%)	2 (5.9)	-	2 (10)	0.230
	SLE, n (%)	1 (2.9)	-	1 (5)	0.403
At the ICU admission	APACHE II score*	23 [18–28]	17 [11–24]	25 [21–30]	0.004
	SOFA score *	7 [5–10]	6 [4–8]	9 [5–10]	0.097
	BVAS*	23 [16–30]	21 [13–29]	24 [16–31]	0.539
	SAPS II*	44 [34–52]	40 [33–44]	49 [37–58]	0.044
	GCS*	14 [11–15]	14 [13–15]	13 [8–14]	0.164
	Days from diagnosis to ICUad *	25 [0–430]	14 [0–588]	33 [0–166]	0.972
	IMV, n (%)	7 (20.6)	2 (14.3)	5 (25)	0.616
	NIMV, n (%)	15 (44.1)	5 (35.7)	10 (50)	0.500
	Hemorrhage, n (%)	29 (85.3)	10 (71.4)	19 (95)	0.966
	· Massive hemoptysis, n (%)	20 (58.8)	7 (50)	13 (65)	0.389
	· GIS bleeding, n (%)	7 (20.6)	1 (7.1)	6 (30)	0.110
	· Hematuria, n (%)	2 (5.9)	2 (14.3)	-	0.086
	Infection, n (%)	32 (94.1)	13 (92.9)	19 (95)	0.797
	· Pneumonia, n (%)	28 (82.4)	11 (78.6)	17 (85)	0.634
	· UTI, n (%)	3 (8.8)	2 (14.3)	1 (5)	0.355
	· CRBSI, n (%)	3 (8.8)	2 (14.3)	1 (5)	0.355
	Sepsis, n (%)	23 (67.6)	9 (64.3)	14 (70)	0.730
	Septic shock, n (%)	10 (29.4)	-	10 (50)	0.014
During ICU stay	Intubation requirement, n (%)	21 (61.8)	6 (42.9)	15 (75)	0.061
	IMV, n (%)	27 (79.4)	7 (50)	20 (100)	0.0001
	NIMV, n (%)	20 (58.8)	9 (64.3)	11 (55)	0.594
	Tracheostomy, n (%)	3 (8.8)	-	3 (15)	0.135
	New onset infection, n (%)	23 (67.6)	6 (42.9)	17 (85)	0.011
	· Pneumonia, n (%)	22 (64.7)	6 (42.9)	16 (80)	0.028
	· UTI, n (%)	4 (11.8)	-	4 (20)	0.079
	· CRBSI, n (%)	2 (5.9)	-	2 (10)	0.230
	New onset sepsis, n (%)	15 (44.1)	1 (7.1)	14 (70)	0.0001
	New onset septic shock, n (%)	14 (41)	3 (21.4)	11 (55)	0.054
	HD requirement, n (%)	22 (64.7)	8 (57.1)	14 (70)	0.447
	CRRT requirement, n (%)	11 (32.4)	-	11 (55)	0.001
	Length of ICU stay (day)*	15 [7-24]	13 [7-20]	17 [7-25]	0.451

*Data are presented as median [interquartile range], n: number; M: male; BVAS: Birmingham Vasculitis Activity Score; APACHE: Acute Physiology and Chronic Health Evaluation; SOFA: Sequential Organ Failure Assessment; GCS: Glasgow Coma Scale; SAPS: Simplified Acute Physiology Score; CRRT: Continuous Renal Replacement Therapy; ICUad: Intensive Care Unit admission; IMV: Invasive mechanical ventilation; NIMV: Noninvasive mechanical ventilation; GIS: Gastrointestinal system; UTI: Urinary tract infection; CRBSI: Catheter-related bloodstream infection; SLE: Systemic lupus erythematosus; AKIN: acute kidney injury network; HD: hemodialysis;

**Table 2:** Disease related features for all AAV patients including survivors and nonsurvivors.

	Parameters, n (%)	All (n = 34)	Survivors (n = 14)	Nonsurvivors (n = 20)	P value
	c–ANCA	24 (70.6%)	10 (71.4%)	14 (70%)	0.986
	p–ANCA	12 (35.3%)	5 (35.7%)	7 (35%)	0.959
	GPA	25 (73.5%)	10 (71.4%)	15 (75%)	0.819
	MPA	9 (26.5%)	4 (28.6%)	5 (25%)	0.819
	BVAS at the diagnosis*	23 [19–29]	20 [12–28]	24 [20–31]	0.089
	Active vasculitis	30 (88.2%)	12 (85.7%)	18 (90%)	0.707
	New-diagnosed AAV	18 (52.9%)	8 (57.1%)	10 (50%)	0.686
	Relapsed AAV	12 (35.3%)	4 (28.6%)	8 (40%)	0.499
Organs/systemsinvolvement	Pulmonary-renal syndrome	22 (64.7%)	9 (64.3%)	13 (65%)	0.966
	Only pulmonary involvement	7 (20.6%)	2 (14.3%)	5 (25%)	0.454
	Cardiovascular system	8 (23.5%)	2 (14.3%)	6 (30%)	0.298
	Central nervous system	8 (23.5%)	2 (14.3%)	6 (%30)	0.295
	Gastrointestinal system	7 (20.6%)	1 (7.1%)	6 (30%)	0.110
	Eye	5 (14.7%)	3 (21.4%)	2 (10%)	0.362
	Skin	5 (14.7%)	1 (7.1%)	4 (20%)	0.305
	Upper airway	5 (14.7%)	2 (14.3%)	3 (15%)	0.955
The Main Reasonsof ICU admission	Respiratory failure due to massive hemoptysis	14 (41.2%)	7 (50%)	7 (35%)	0.478
	Septic shock	9 (26.5%)	-	9 (45%)	0.027
	Sepsis without shock and impaired consciousness	4 (11.8%)	3 (21.4%)	1 (5%)	0.436
	Heart failure and pulmonary edema	3 (8.8%)	2 (14.3%)	1 (5%)	0.666
	Gastrointestinal bleeding and shock	2 (5.9%)	1 (7.1%)	1 (5%)	0.797
	Kidney failure and pulmonary edema	2 (5.9%)	1 (7.1%)	1 (5%)	0.797

*Data are presented as median [interquartile range] n: number; AAV: Antineutrophil Cytoplasmic Antibody Associated Vasculitis; c-ANCA: Cytoplasmic Antineutrophil Cytoplasmic Antibodies; p-ANCA: Perinuclear Antineutrophil Cytoplasmic Antibodies; GPA: Granulomatosis with polyangiitis; MPA: Microscopic polyangiitis; BVAS: Birmingham Vasculitis Activity Score;

**Table 3:** Some laboratory data for all AAV patients including survivors and nonsurvivors.

Parameters	All (n = 34)	Survivors (n = 14)	Nonsurvivors (n = 20)	P value
Max. sAST (U/L)*	48 [35–282]	38 [32–66]	103 [40–912]	0.016
Max. sALT (U/L)*	60 [22–193]	39 [18–63]	124 [48–638]	0.014
Max. sT.Bil.(mg/dL)*	1.7 [1–4.2]	1.2 [0.6–1.7]	3.8 [1.4–7.4]	0.005
Max. sLactat (mmol/L)*	4 [2.2–9.9]	2.1 [1.4–3.8]	6 [3.7–15.5]	0.001
Max. sCRP (mg/L)*	151 [32–207]	60 [20–200]	175 [101–233]	0.041
Max. sPRC (ng/mL)*	19.5 [2.2–73]	3 [0.2–23]	29 [4.8–82]	0.024
Min. bWBC (x103/μL)*	3.4 [1.2–6.2]	4.4 [3.3–7.6]	2.2 [0.6–4.1]	0.027
Min. bPlatelet (x103/μL)*	38 [9.5–86]	78 [67–203]	12.4 [5.7–31.5]	0.0001
Min. arterial pH*	7.13 [7.0–7.2]	7.2 [7.1–7.3]	7.07 [6.9–7.1]	0.0001

*Data are presented as median [interquartile range], s: serum; b: blood; Max: Maximum; Min: Minimum; AAV: Antineutrophil Cytoplasmic Antibody Associated Vasculitis; CRP: C-Reactive Protein; PRC: Procalcitonin; AST: Aspartate Aminotransferase; ALT: Alanine Aminotransferase; T.Bil: Total Bilirubin; PH: Power of Hydrogen;

**Table 4:** Immunosuppressive therapies in all AAV patients including survivors and nonsurvivors.

	Parameters	All (n = 34)	Survivors (n = 14)	Nonsurvivors (n = 20)	P value
Before ICU admission	Methylprednisolone, n (%)	26 (76.5)	9 (64.3)	17 (85)	0.167
	Cyclophosphamide, n (%)	20 (58.8)	7 [50]	13 (65)	0.389
	Rituximab, n (%)	6 (17.6)	3 (21.4)	3 (15)	0.634
	Plasmapheresis, n (%)	16 (47.1)	4 (28.6)	12 (60)	0.075
	Total seans*, count	5 [4–7]	5 [2–9]	6 [4–7]	0.691
During ICU stay	Methylprednisolone, n (%)	31 (91.2)	13 (92.9)	18 (90)	0.776
	Cyclophosphamide, n (%)	7 (20.6)	5 (35.7)	2 (10)	0.072
	Rituximab, n (%)	1 (2.9)	-	1 (5)	-
	Plasmapheresis, n (%)	18 (52.9)	8 (57.1)	10 (50)	0.686
	Total seans*, count	4 [3–5]	5 [3–6]	3 [3–5]	0.256

*Data are presented as median [interquartile range] AAV: Antineutrophil Cytoplasmic Antibody Associated Vasculitis; n: number; ICU: Intensive Care Unit

**Table 5:** New onset infections related microorganisms during ICU stay in all AAV patients including survivors and nonsurvivors.

Related microorganisms	All (n = 34)	Survivors (n = 14)	Nonsurvivors (n = 20)	P value
Acinetobacter baumannii, n (%)	10 (29.4)	3 (21.4)	7 (35)	0.399
Escherichia coli, n (%)	8 (23.5)	2 (14.3)	6 (30)	0.288
Klebsiella pneumonia, n (%)	4 (11.8)	2 (14.3)	2 (10)	0.703
Enterococcus spp, n (%)	4 (11.8)	-	4 (20)	0.075
Candida spp, n (%)	4 (11.8)	-	4 (20)	0.075
Stenotrophomonas maltophilia, n (%)	2 (5.9)	-	2 (10)	0.223
Pseudomonas aeruginosa, n (%)	2 (5.9)	1 (7.1)	1 (5)	0.794

AAV: Antineutrophil Cytoplasmic Antibody Associated Vasculitis; n: number; ICU: Intensive Care Unit

**Table 6:** Logistic regression analysis for the determination of independent risk factors for ICU mortality in AAV patients.

Parameters	P value	Wald	Exp (B)	CI 95% (Confidence Interval)
APACHE II score	0.038	4.295	1.231	1.011–1.498
SAPS II score	0.895	0.017	0.993	0.893–1.104
SOFA score	0.949	0.004	0.988	0.681–1.434
BVAS at the diagnosis	0.473	0.514	1.034	0.944–1.132
Serum ALT level	0.600	0.275	1.005	0.988–1.022
Serum Platelet count	0.473	0.127	1.034	0.944–1.132

AAV: Antineutrophil Cytoplasmic Antibody Associated Vasculitis; APACHE: Acute Physiology and Chronic Health Evaluation; SAPS: Simplified Acute Physiology Score; SOFA: Sequential Organ Failure Assessment; BVAS: Birmingham Vasculitis Activity Score; ICUad: ALT: Alanine aminotransferase

**Figure:**
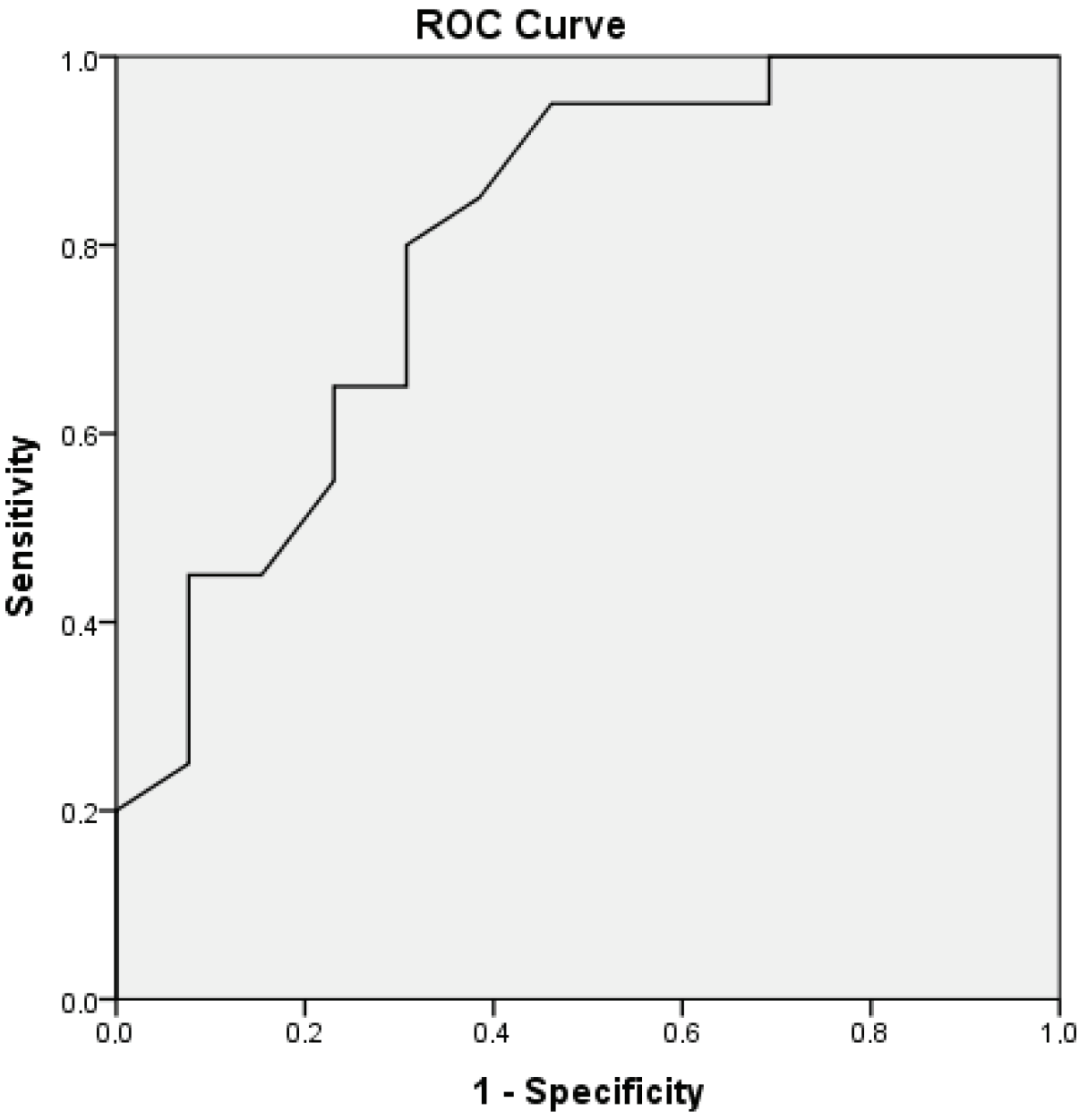
ROC-Curve analysis of APACHE II score for ICU mortality (AUC= 0.8, P = 0.004). The cutoff value of APACHE II score = 20.5 (80% sensitivity, 70% specificity, LR = 2.6)

## 4. Discussion

ANCA associated vasculitis patients are accepted to ICUs due to the initial presentation of the vasculitis or the result of progressive primary or recurrent/refractory disease or the result of complications of immunosuppressive treatment [15,16]. In our study, most of the patients (88.2%) had an active disease of AAV at the ICU admission (newly diagnosed or relapsing disease). Also, the majority of the study patients were admitted to our ICUs with hemorrhage (85.3% – especially massive hemoptysis) and with sepsis (67.6%).

In general, it is known that the presence of an underlying rheumatologic disease [17] or vasculitic disease in ICU patients has a negative effect on their outcome. This reality is also true for AAV patients. Previous studies have found highly heterogeneous results related to ICU mortality of AAV patients. For example, Demiselle et al. reported that the mortality rate of AAV patients with acute vasculitis manifestation was 15.5% [10]. In a similar study that included the patients of small vessel vasculitis, the mortality rate was found 16% in the ICU follow up period [13]. In other studies, the ICU mortality of the AAV patients was found as 33, 39, and 60%, respectively [4,12,18]. These variable mortality rates were probably related to the heterogeneity of included patients. In our study, we found that the ICU mortality rate of AAV patients was 58.8%. It was slightly higher than the previous studies. Reasons for the higher mortality observed in our patient group might be the presence of high rate of active vasculitis, renal and pulmonary insufficiency related with active vasculitis, high requirements of mechanical ventilation, and renal replacement therapy and presence of high rate of infection and sepsis at the ICU admission or during the ICU stay. Also, our departments are referral centers for patients with vasculitis. Patients with worse conditions or those who do not respond adequately to standard therapy, in particular, are referred to our centers. This situation may have led to higher mortality rates in our study. In our opinion and experience, this result is important because this group of diseases is associated with severe complications that may lead to fatal outcomes.

As for the role of prognostic scores (APACHE II, SOFA, SAPS II, etc.) in predicting the outcome of patients with AAV admitted to the ICU, our results confirmed the findings of previous studies. Both APACHE II and SAPS II scores were significantly higher in nonsurvivors than in survivors. This finding seems to be consistent with the literature. In the study of Frausova et al. [4], there was a statistically significant difference in the APACHE II score for ICU mortality. Also, in the studies of Demiselle et al. [10] and Kimmoun et al. [13], there were statistically significant differences for SAPS II scores according to ICU mortality. In the study of Cruz et al. [12], there were statistically significant differences in APACHE II and SAPS II scores according to ICU mortality. But SAPS II and APACHE II scores, which are nonspecific ICU severity scores assessed at admission, were generally found to be predictive of ICU mortality in all types or all groups of ICU patients. Nevertheless, we found that if the APACHE II score was equal or higher than 20.5 at the time of ICU admission, the chance of survival decreased significantly in our AAV patients. Burkhardt et al. reported similar results in patients with Wegener’s granulomatosis [19].

Some studies have shown a relationship between vasculitic activity and long-term and short-term (such as in ICU mortality) prognosis [20, 21]. BVAS score has been used most frequently in these studies. BVAS has become the current standard assessment tool for scoring activity in AAV. But in our study, we did not find any correlation between vasculitis disease activity (assessed by BVAS) and ICU mortality. BVAS (neither BVAS on the first ICU day nor BVAS at vasculitis diagnosis) was not associated with ICU mortality in our study. This finding is supported by other studies investigating ICU mortality of AAV patients [4,10–13]. It is known, however, that BVAS was designed to assess vasculitic patients prospectively, and the retrospective analysis might have underestimated the score.

Apart from these prognostic scoring systems, in the literature, some factors have been stated to have prognostic importance in the short-term mortality of the AAV patients. These factors can be listed as the presence of infections, requirements of vasopressors, mechanical ventilation and blood transfusion, usage of cyclophosphamide, and renal failure [2,10]. In our study, new onset ICU infections, shock and vasopressor requirements, thrombocytopenia and platelet transfusion requirements, and liver dysfunction can be listed as factors that were significantly related to ICU mortality of AAV patients in univariate analysis.

Several limitations must be considered when interpreting the results of this study, which include its retrospective nature and small study size. The retrospective analysis of the data might have influenced the results. Furthermore, although our departments serve as referral centers, the number of patients in our study is relatively small due to the low incidence and prevalence of the disease in general. Because this study covers a period of 10 years, the difference in treatment approach and technology over the years may have affected patient outcomes. As this is a multicenter study, there may be differences between the treatment approaches of the centers. Even so, our study is the first multicenter study from Turkey to highlight the clinical predictors of ICU mortality in patients with ANCA associated vasculitis.

In conclusion, this study is the first multicenter study from Turkey, which included patients who needed ICU admission for diagnosis and treatment of AAV activation or complications. The study tried to reveal factors affecting ICU mortality in patients with ANCA associated vasculitis. APACHE II score was found to be valuable for the prediction of ICU mortality in this patient group.

## Acknowledgement

No financial support or grant was received for this study.

## Informed Consent

This study was approved by the Local Ethics Committee of Gazi University (date: March 26, 2018 and issue: 234).
